# Analysis of endophytic and rhizosphere bacterial diversity and function in the endangered plant *Paeonia ludlowii*

**DOI:** 10.1007/s00203-020-01882-3

**Published:** 2020-04-20

**Authors:** Yazhou Lu, Erhao Zhang, Mingsheng Hong, Xiu Yin, Hao Cai, Lei Yuan, Fang Yuan, Lianqiang Li, Kentian Zhao, Xiaozhong Lan

**Affiliations:** 1 Research Institute of Plateau Ecology, Tibet Agriculture and Animal Husbandry University, Nyingchi, 860000 Tibet China; 2 Medicinal Plants Joint Research and Development Centre, Tibet Agriculture and Animal Husbandry College-Southwest University, Nyingchi, 860000 Tibet China; 3 Food Science College, Tibet Agriculture and Animal Husbandry University, Nyingchi, 860000 Tibet China; 4 Department of Resources and Environment, Tibet Agriculture and Animal Husbandry University, Nyingchi, 860000 Tibet China; 5 Key Laboratory of Southwest China Wildlife Resources Conservation, China West Normal University, Ministry of Education, Nanchong, 637009 Sichuan China

**Keywords:** Endophytic bacterial community, Illumina MiSeq sequencing, *Paeonia ludlowii*

## Abstract

*Paeonia ludlowii* is indigenous to Tibet and has an important ecological and economic value in China. In Tibet, *P. ludlowii* has been used in folk medicine with relative success. Plant microbial endophytes play an important role in plant growth, health and ecological function. The diversity of endophytic bacteria associated with *P. ludlowii* remains poorly understood. In this study, the structure of the endophytic bacterial communities associated with different tissues, including fruits, flowers, leaves, stems, and roots, and rhizosphere soils was analyzed with Illumina MiSeq sequencing of bacterial 16S rDNA. A total of 426,240 sequences and 4847 operational taxonomic units (OTUs) were obtained. The OTUs abundance of roots was higher than that of other tissues; however, the OTUs abundance was similar among different deep soil samples. In the plant tissues, Cyanobacteria was the most abundant bacterial phylum, followed by Proteobacteria; however, the most abundant phyla were Proteobacteria and Acidobacteria in soil samples from three different layers. In addition, the diversity and richness of the microorganisms in the soil were very similar to those in roots but higher than those in other tissues of *P. ludlowii*. Predictive metagenome analysis revealed that endophytic bacteria play critical functional roles in *P. ludlowii*. This conclusion could facilitate the study of the ecological functions of endophytic bacteria and their interactions with *P. ludlowii* to analyze the reasons why this important medicinal plant is becoming endangered.

## Introduction

The Tibetan peony, *Paeonia ludlowii*, is an endemic species to the Himalayan-Hengduan Mountains; it is renowned as a medicinal plant that reduces inflammation (Liu et al. 2017; Lou et al. 2017) and is only distributed in a small area of southern Tibet in western China (Hao et al. 2014). *P. ludlowii*, an important rare species with high economic, medicinal and ornamental value (Zhang et al. 1997), is on the verge of extinction as a result of its low germination percentage and long germination period and has been listed in the Endangered Species Red Book of China. Endophyte studies might provide a way to improve seed germination. A large number of articles have documented that endophytes can facilitate interim germination and improve the germination rate, length of coleoptiles and radicles, seedling dry weight, stress resistance and early plant development (Gao and Shi 2018; Li et al. 2017; Hubbard et al. 2014); however, the endophytes of *P. ludlowii* have not been explored.

Endophytes are nonpathogenic microbes that reside in healthy plant tissues and benefit both the plants and the microbes. Bacterial endophytes play important roles in plant growth, health and ecological function, conferring certain benefits to plants (Lumactud and Fulthorpe 2018). A growing body of literature has reported that bacterial endophytes promote plant growth, improve plant health, enhance plant tolerance to stress and provide many additional benefits (Azevedo et al. 2000; Hardoim et al. 2008; Glick and Stearns, 2011; Mitter et al. 2017). Endophytic bacteria have received increasing research attention because of their potential biological functions in recent years (Compant et al. 2005). Thus, understanding the community and diversity of endophytic bacteria in plant tissues is imperative.

With the development of society, endophytic bacteria of medicinal plants have gained more attention as a consequence of their substantial potential to synthesize numerous novel pharmaceutical compounds, such as antifungal, antibiotic, anticancer, antiviral, and immunosuppressant compounds (Golinska et al. 2015). Therefore, fully understanding the community and diversity of endophytic bacteria may help to exploit the potential of medicinal plants (El-Deeb et al. 2012). It is well known that the application of medicinal plants has a long history in China, and over 500 medicinal plants have been listed in the Chinese Pharmacopeia to date; hence, medicinal plants are a very important and relatively untapped source of pharmaceutical compounds (Golinska et al. 2015). Because of the importance of endophytic bacteria in plants, studying these endophytic bacteria is of immense significance.

In this study, we aimed to investigate the bacterial endophyte diversity and community distribution in *P. ludlowii* in the Himalayan-Hengduan Mountains and provided a potentially valuable strategy for the propagation of *P. ludlowii* by analyzing the endophytic bacteria in the different plant tissues and rhizosphere soils of this species.

## Materials and methods

### Sampling and treatment

*P. ludlowii* samples were collected from the Himalayan-Hengduan Mountains in MiRui Township (29º32′43.96′′N, 94º38′33.03′′E), Nyingchi City, Tibet, China. The tissues included fruit pods, flowers, leaves, stems, and roots. Rhizosphere soil was also collected (the soil was divided into upper, middle and lower layers at depths of 0–10 cm, 10–20 cm and 20–30 cm, respectively) during the *P. ludlowii* fruiting stage (June 5, 2018). The plant tissues and soil samples were randomly selected from 10 tree peonies and mixed. All types of samples were taken in triplicate and collected into sterile plastic bags and processed within 24 h. Plant samples were washed with water. Then, each tissue was surface-sterilized with 75% ethanol for 30 s and 10% sodium hypochlorite for 5 min. The samples were then washed with sterile PBS solution. Finally, the success of the sterilization was tested by wiping sterilized tissues across the surface of a Petri plate with Luria–Bertani (LB) medium to ensure the absence of bacterial growth. The soil samples were dried at 105 °C for 24 h.

### DNA extraction, PCR amplification of the 16S rDNA region and sequencing

Approximately 2 g of sterilized plant tissues were frozen in liquid nitrogen and ground to a fine powder in a sterilized and precooled mortar. Total DNA was extracted using a bacterial DNA extraction kit (Omega, D3350-01, USA). The soil DNA was extracted using a soil DNA isolation kit (Sigma, DNB100-50RXN, USA). Three biological replicates were performed for each tissue and soil sample, and the quantity and quality of DNA were measured with a NanoDrop One spectrophotometer (Thermo Fisher Scientific, USA) and agarose gel electrophoresis (AGE).

The bacterial 16S rRNA gene was amplified using the following primer pairs: forward, 5′- ACTCCTACGGGAGGCAGCA, and reverse, 3′- GGACTACHVGGGTWTCTAAT (Masoud et al. 2011). The 25 μL PCR mixture contained 2.5 μL 10 × reaction buffer, 0.2 μL rTaq DNA polymerase (Invitrogen, USA), 2 μL 5 mmol/L dNTPs, 1 μL 5 μmol/L forward and reverse primers, 10 ng template DNA, and water to a final volume of 25 μL. PCR amplification was performed under the following conditions: initial denaturation at 94 °C for 5 min; 35 cycles of 94 °C for 30 s, 60 °C for 30 s, and 72 °C for 1 min; a final extension at 72 °C for 10 min; and a final hold at 4 °C until further use. The PCR products were measured on 2% agarose gels containing Gold view and then purified with a DNA gel extraction kit (Tiangen, China). The purified DNA samples were subjected to high-throughput sequencing at the Beijing Genomics Institute (BGI) using Illumina technology. Illumina sequence reads were deposited under the NCBI SRA accession number PRJNA600148.

### Sequence processing and analysis

Sequence processing and quality filtering, denoising, trimming, and merging of raw paired-end FASTQ files were performed using an improved dual-indexing approach and FLASH software to obtain clean tags (Fadrosh et al. 2014; Magoc and Salzberg 2011). The chloroplast and mitochondrial DNA was eliminated from further analyses. The obtained clean tags were assigned into operational taxonomic units (OTUs) using USEARCH (v7.0.1090) at 97% identity clustering (Edgar et al. 2013). The observed species index, Chao index, ACE index, Shannon index and Simpson index were determined using R software v3.1.1, which reflected the alpha diversity (Schloss et al. 2009). The Shannon diversity and Chao1 richness were determined and principal coordinate analysis (PCoA) was performed using QIIME (v1.80) (Caporaso et al. 2010). Heatmap and ternary plots were analyzed using R v3.1.1. All data were statistically analyzed using one-way analysis of variance. Tukey’s honestly significant difference test was used to separate means at *p* = 0.05.

## Results

### Analysis of sequencing data and bacterial community diversity

**Fig. 1:**
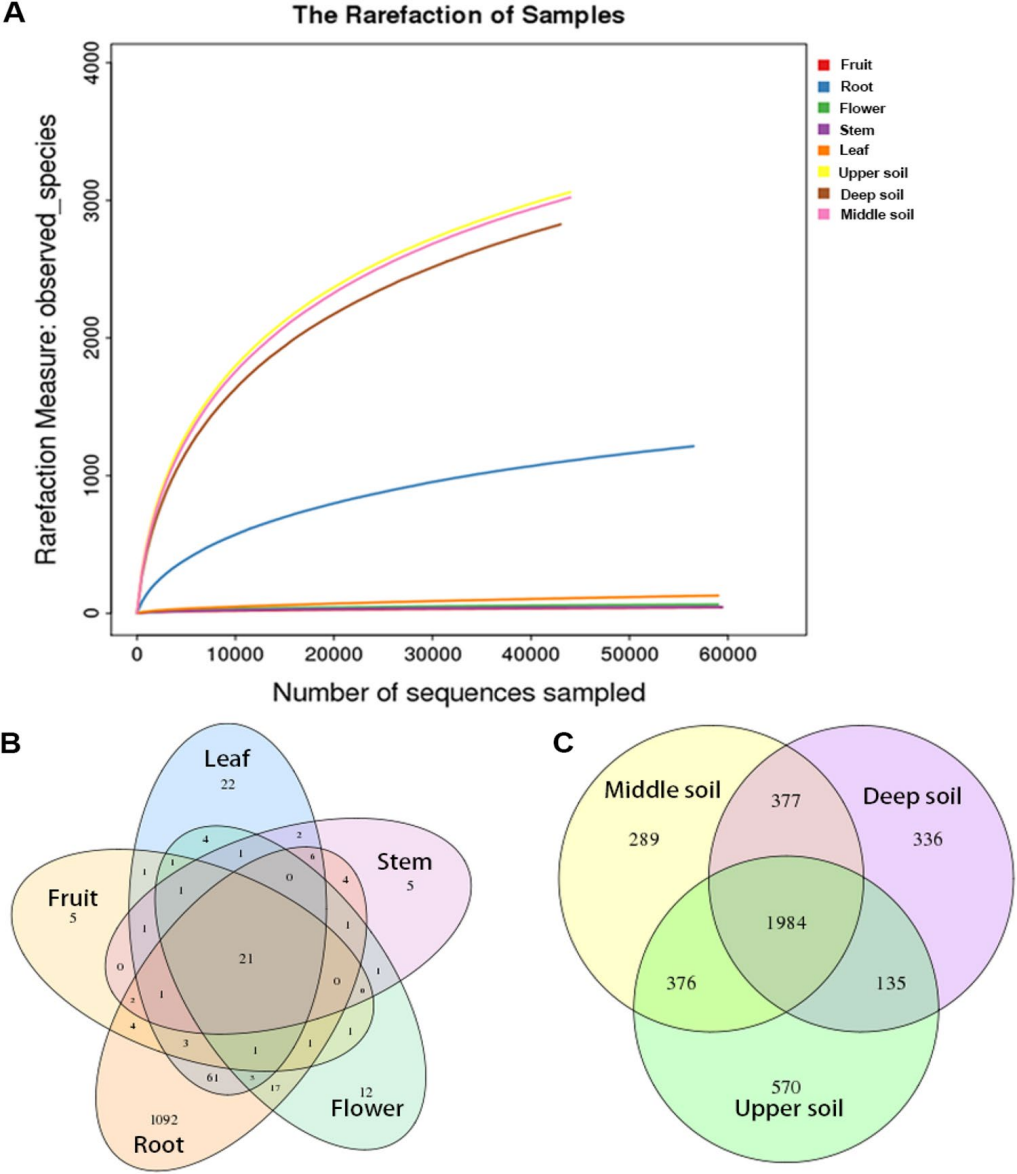
**a** Rarefaction curves for bacterial endophyte OTUs in different tissues and soils of *P. ludlowii*. **b** Venn diagrams showing the number of shared and unique OTUs in different tissue and soil samples

A total of 426,240 high-quality tag sequences were obtained with an average length of 250 bp across fruit, flower, leaf, stem, root, and soil samples after sequence denoising and quality filtering. The numbers of obtained sequences ranged from 43,322 to 59,410, with an average of 53,280 ± 7763 (mean ± SD) sequences across all eight samples. All quality-filtered sequences were clustered into 4847 OTUs at a 97% similarity level. The number of clustered OTUs varied from 43 to 3065. All rarefaction curves gradually saturated with increasing sequencing quantity in all 8 samples that covered the entire group. The results of rarefaction curves showed that OTU abundance was diverse in different tissue and soil samples. The numbers of OTUs were significantly higher in the soil samples than in the tissue samples (upper 3065, middle 3026, deep 2832). The root samples revealed a higher number of OTUs (1217), while fruit, stem and flower tissues showed lower richness, with 43, 46 and 65 OTUs, respectively. The different tissues shared 21 OTUs of the total 1500 OTUs (Fig. 1b), while 1984 OTUs of the total 8923 OTUs were shared among different soil samples (Fig. 1c).

Among the types of tissue, the highest richness and diversity of the bacterial community were found in roots, and the richness and diversity of the bacterial community were the lowest in stems (Table 1). In the rhizosphere soils, the upper soil and middle soil had the same diversity, whereas the richness of the bacterial community in the upper soil was higher than that in the middle soil, and the lowest richness and diversity were observed in the deep soil (Table 1).

**Table 1:** The number of OTUs and *alpha* diversity of endophytic bacteria in *P. ludlowii* and rhizosphere soil

Sample	Number of sequences	Sobs	*Alpha* diversity				
			Chao1	Ace	Shannon	Simpson	Coverage (%)
Fruit	59,410	43	64.38	116.65	0.52	0.704	99.97
Root	56,992	1217	1652.45	1678.53	2.08	0.497	99.26
Flower	59,385	65	96.63	91.09	0.43	0.776	99.96
Stem	59,400	46	51.5	57.87	0.44	0.781	99.98
Leaf	59,168	129	249.75	400.05	0.64	0.666	99.88
Upper soil	44,305	3065	3881.45	3869.02	6.49	0.007	98.04
Deep soil	43,322	2832	3735.96	3706.1	6.11	0.01	97.98
Middle soil	44,258	3026	3854.18	3837.09	6.38	0.007	98.01

### Microbial taxonomic analysis at the phylum and class levels

The tag numbers of each taxonomic rank from phylum to species or each OTU in different samples were summarized with QIIME. The classification of sequences also demonstrated bacterial community differences in tissues and rhizosphere soils at the phylum level. In this study, the sequences were clustered into 43 bacterial phyla. The bacterial composition varied among the different tissues; for example, the number of bacterial phyla was higher in roots (28 phyla) than in fruits (8 phyla), while the phyla were similar in different rhizosphere soil samples from the deepest layer. It was obvious that Cyanobacteria was the most dominant phylum, accounting for 70.3–88.0% of all bacterial sequences in the different tissues, followed by Proteobacteria (11.9–19.6%). The abundance of each phylum varied in the different tissues. Actinobacteria, Verrucomicrobia, Bacteroidetes, Acidobacteria, and Firmicutes were also predominant phyla (> 1% relative abundance) in the root tissues, contributing to 1.2–3.4% of the relative abundance, whereas the abundance of these five phyla was less than 1% in other tissues. These results demonstrated that the relative abundance of bacterial communities in the roots was much higher than that in other tissues.

**Fig. 2:**
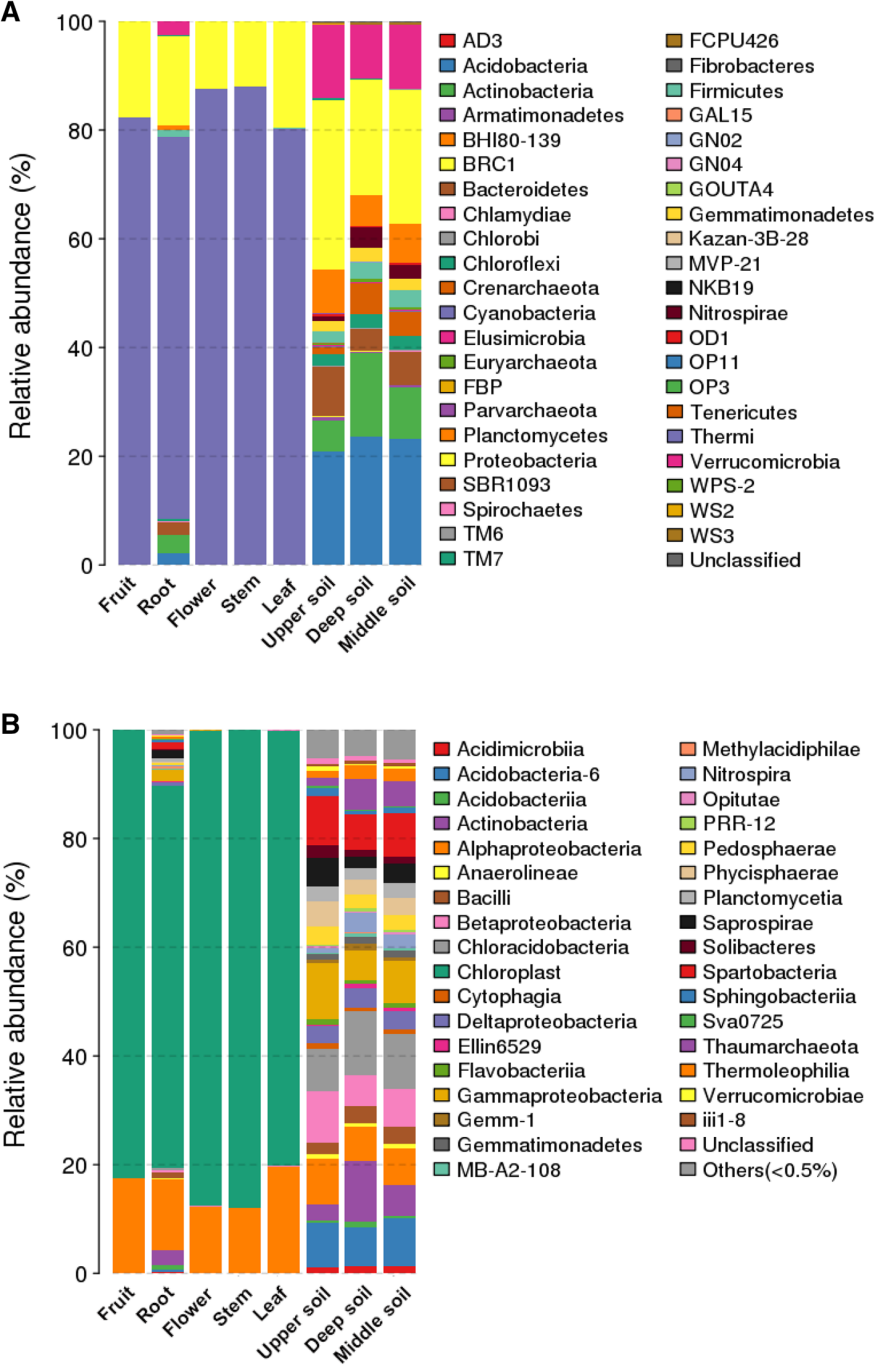
The relative abundances of bacterial communities at (**a**) the phylum level and (**b**) the class level in the different samples

The abundance of bacterial communities was significantly higher in the rhizosphere soil than in the tissues. Proteobacteria and Acidobacteria were the dominant phyla, accounting for 21.2–31.1% and 20.9–23.6% of all bacterial sequences, respectively, followed by Actinobacteria (5.7–15.4%), Bacteroidetes (4.0–9.0%), Crenarchaeota (1.3–5.7%), Firmicutes (2.2–3.1%), Gemmatimonadetes (1.8–2.6%), Planctomycetes (5.6–8.0%), and Verrucomicrobia (9.9–13.6%) (Fig. 2a). At the class level, species in Cyanobacteria and Alphaproteobacteria showed virtually absolute dominance, representing 70.3–88.0% and 11.9–19.5% of the relative abundance, respectively. However, Actinobacteria (2.7%), Bacilli (1.1%), Gammaproteobacteria (2.1%), Saprospirae (1.6%), and Spartobacteria (1.2%) were also predominant (> 1% relative abundance) in the root tissues, whereas their abundance was less than 1% in other tissues. In the rhizosphere soil, Alphaproteobacteria, Acidobacteria-6, Betaproteobacteria, Chloracidobacteria, Gammaproteobacteria, and Spartobacteria were the predominant groups, comprising approximately 48.2% of the relative abundance at the class level (Fig. 2b), and the abundances of the classes exceeding 1% in every tissue are shown in Table 2. These results showed that the relative abundance of bacterial communities in the rhizosphere soil was significantly higher than that in the plant tissues.

**Table 2:** The relative abundances of the classes exceeding 1% in all sample

The relative abundance (>1%)	Fruit	Root	Flower	Stem	Leaf	Upper soil	Deep soil	Middle soil
Acidimicrobiia	/	2.69	/	/	/	1.21	1.36	1.38
Acidobacteria-6	/	/	/	/	/	8.09	7.19	8.75
Acidobacteriia	/	/	/	/	/	/	1.01	/
Actinobacteria	/	/	/	/	/	2.91	11.11	5.59
Alphaproteobacteria	17.51	13.00	12.29	11.94	19.51	8.33	6.41	6.74
Bacilli	/	1.09	/	/	/	2.23	3.01	3.15
Betaproteobacteria	/	/	/	/	/	9.32	5.82	6.92
Chloracidobacteria	/	/	/	/	/	7.85	11.83	10.20
Cytophagia	/	/	/	/	/	1.15	/	/
Deltaproteobacteria	/	/	/	/	/	2.97	3.52	3.19
Gammaproteobacteria	/	2.14	/	/	/	10.34	5.45	7.81
Gemm-1	/	/	/	/	/	/	1.27	/
Gemmatimonadetes	/	/	/	/	/	1.12	1.25	1.15
Pedosphaerae	/	/	/	/	/	3.20	2.61	2.70
Phycisphaerae	/	/	/	/	/	4.62	2.63	3.15
Planctomycetia	/	/	/	/	/	2.79	2.22	2.93
Saprospirae	/	1.60	/	/	/	5.34	2.04	3.41
Solibacteres	/	1.18	/	/	/	2.23	1.17	1.42
Spartobacteria	/	/	/	/	/	8.97	6.60	7.99
Sphingobacteriia	/	/	/	/	/	1.48	/	1.01
Thaumarchaeota	/	/	/	/	/	1.32	5.71	4.49
Thermoleophilia	/	/	/	/	/	1.39	2.36	2.31

### Bacterial community composition analysis at the family and genus levels

**Fig. 3:**
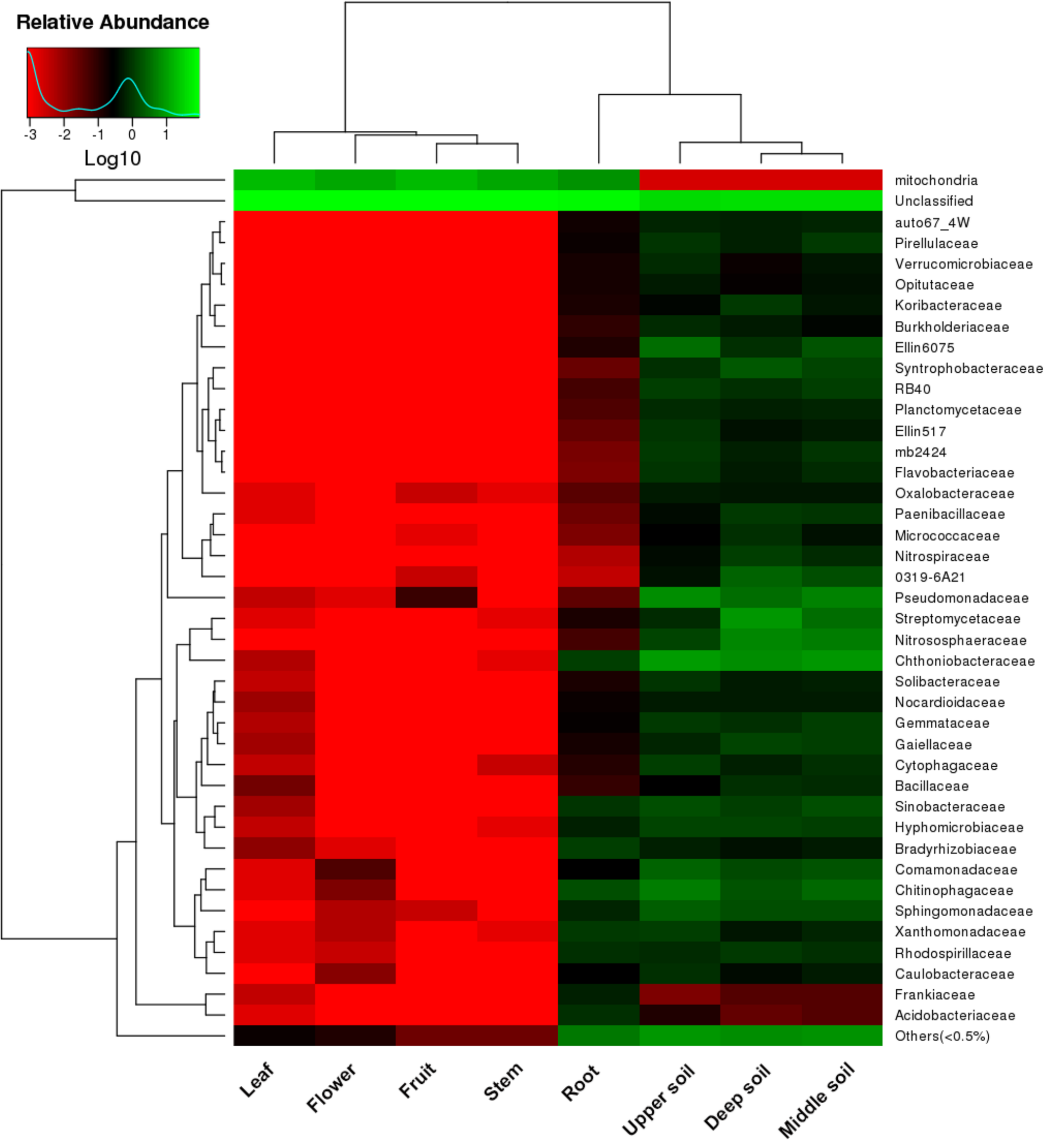
Heatmap analysis of the bacterial composition at the family level for all sample types. The clustering indicates the similarity of certain species among different samples. The heatmap colors from red to green represent the relative abundances from low to high

The heatmap analysis at the family level revealed that bacterial communities were classified into 40 families, which were significantly different between the plant tissues and rhizosphere soils (Fig. 3). The overall bacterial composition of the families differed significantly in the different samples. Chitinophagaceae was the most dominant family in the different plant tissues, accounting for 0.02–1.6% of the relative abundance. However, most of the sequencing data from the plant tissues, accounting for 76.4–88.0% of the total relative abundance, were not clustered into families, which resulted in a lower relative abundance in plant tissues, with the exception of the root tissues. These results demonstrated that the distribution of bacterial communities was much higher in root tissues than in other tissues. In the rhizosphere soil, the distribution and composition of bacterial communities were similar; the dominant families were Chthoniobacteraceae and Pseudomonadaceae, accounting for 6.6–9.0% and 3.4–6.8% of the relative abundance, respectively, followed by Chitinophagaceae (1.8–4.9%), Comamonadaceae (1.4–2.6%), Hyphomicrobiaceae (1.2–1.3%), Nitrososphaeraceae (1.3–5.7%), Sinobacteraceae (1.1–1.7%), and Sphingomonadaceae (1.6–2.4%).

**Fig. 4:**
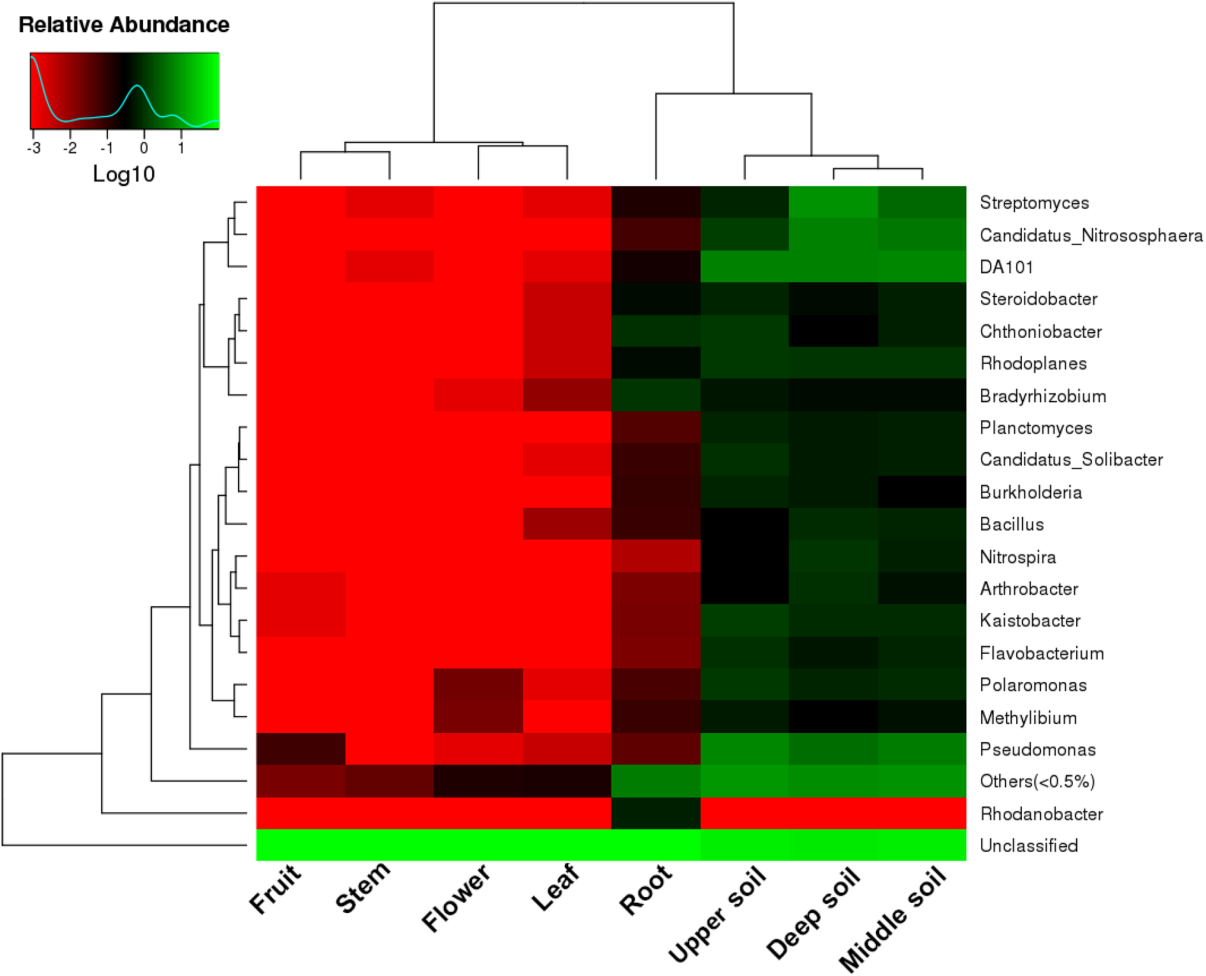
Heat map analysis of the bacterial composition at the genus level for all sample types. The clustering indicates the similarity of certain species among different samples. The heat map colors from red to green represent the relative abundances from low to high

The sequences were classified into 19 different genera, and the heatmap analysis revealed that Rhodanobacter was a genus specific to root tissues (Fig. 4). The distribution of each genus was significantly different across all sample types; for example, there were only three genera and 4 genera in fruit and flower tissues, respectively. DA101 and Pseudomonas were highly abundant in the rhizosphere soil, followed by Candidatus Nitrososphaera and Rhodoplanes. These results showed that the relative abundance of bacterial communities was much higher in the soil than in the plant tissues; nevertheless, the relative abundance of bacterial groups in the root tissues was the highest among the plant tissues, followed by leaves. The clustering analysis indicated that there were more similarities in the plant tissues, except for the roots, and the species compositions of the roots and soil were more similar at the family and genus levels (Figs. 3, 4).

### Comparative analysis of the bacterial communities in the different samples

**Fig. 5:**
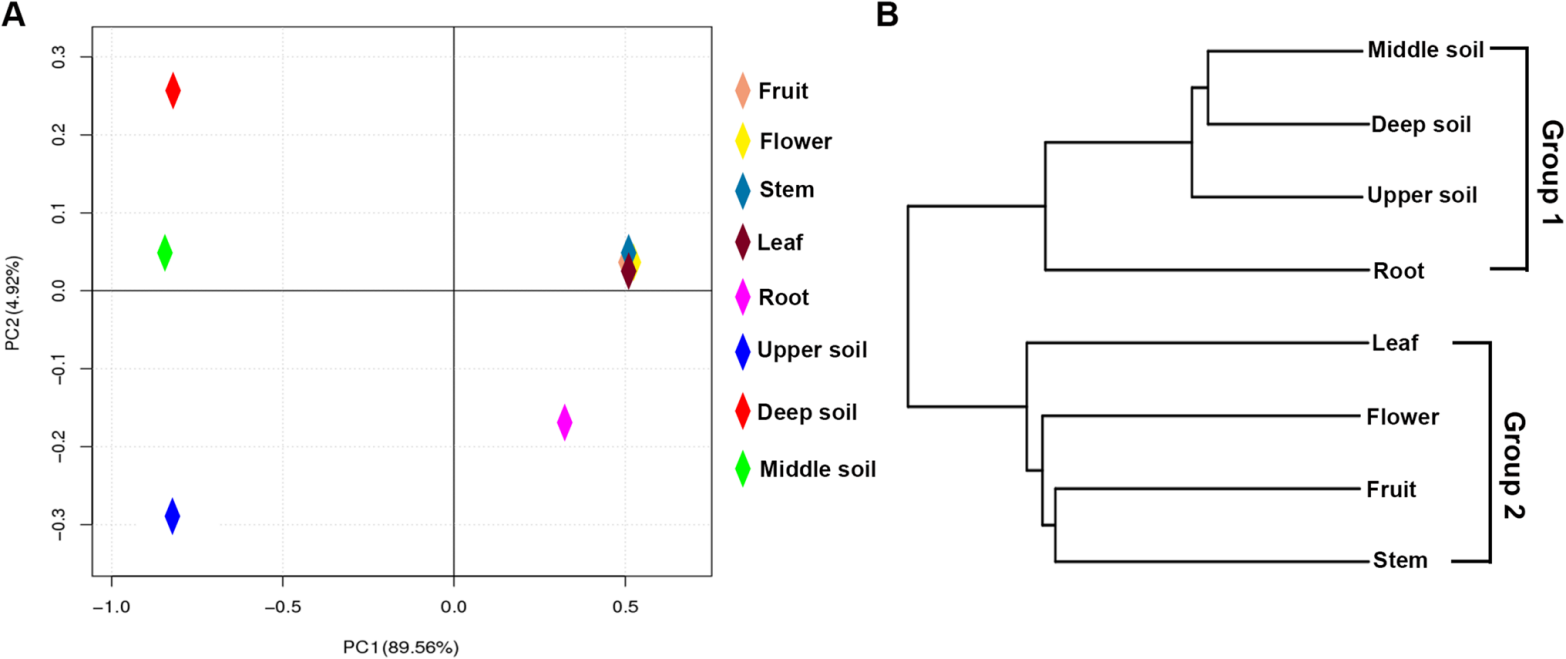
Beta diversity analysis; **a** principal coordinate analysis (PCoA) based on the relative abundance of bacterial OTUs. **b** The complete linkage clustering (CLC) of the bacterial communities in different samples based on unweighted UniFrac distances

To display the differences in OTUs composition in the different samples, PCA was used to illustrate that the bacterial communities in different tissues and soils formed individual clusters (Fig. 5a). The PCA indicated that the community structures were similar in fruit, flower, stem and leaf tissues; in contrast, there were significant differences in community structures among the root tissues and soils, which showed that the roots and soils had their own unique bacterial community structures (Fig. 5a). The results of complete linkage clustering (CLC) tree analysis were similar to those of the PCA (Fig. 5b). In the plant tissues, the roots and other tissues were separated into two different clusters, whereas the root and soil samples were classified into the same group (Fig. 5b). These results indicated that the microbiota in the root samples were largely different from those in other samples.

### Predictive metagenome analysis

**Fig. 6:**
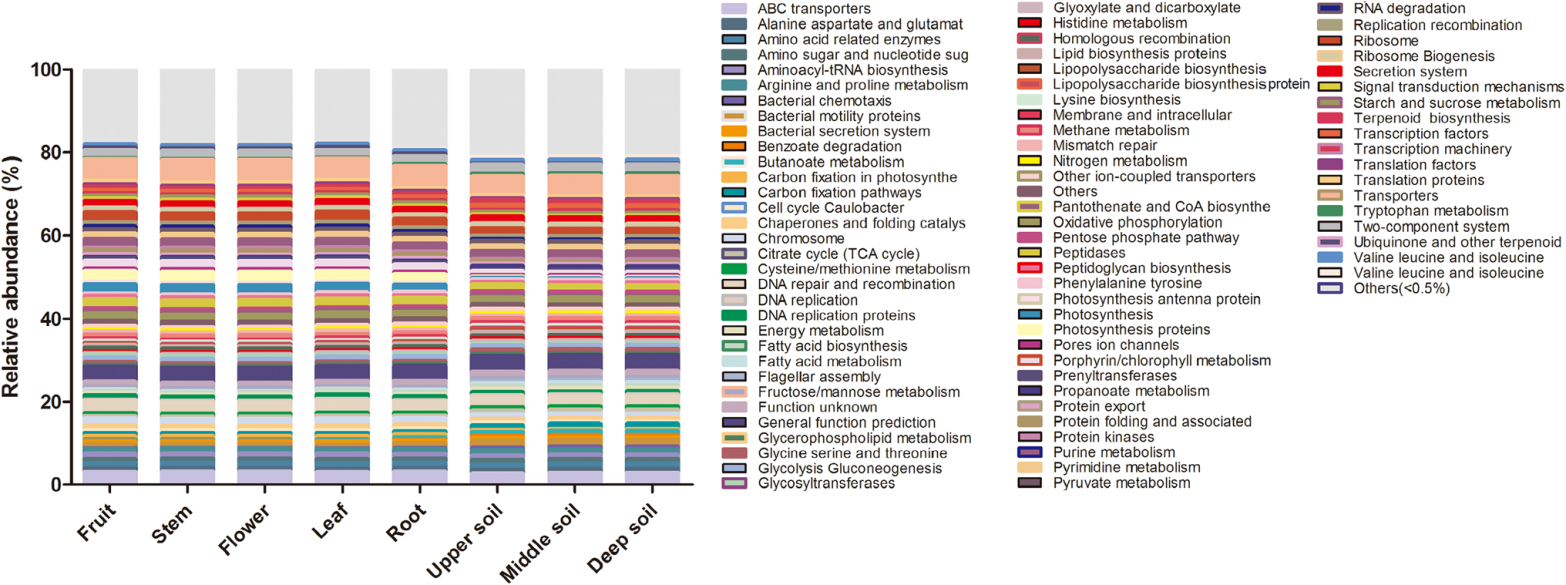
Gene profiles of the bacterial community in *P. ludlowii* tissues and rhizosphere soil predicted using PICRUSt

The PICRUSt approach was used to perform functional classification with the KEGG Orthology (KO) database, and 6 of the level 1 KO groups were found according to their predicted metagenomes, which were involved in cellular processes, environmental information processing, genetic information processing, metabolism, organismal systems and, to a lesser extent, human diseases. A total of 81 levels 3 KO groups were found according to the predicted metagenomes. The major gene families were those relating to ABC transporters, DNA repair and recombination proteins, general function prediction, purine metabolism, peptidases, photosynthesis proteins, ribosomes and transporters (Fig. 6). The relative abundance was similar in the whole tissue and soil samples (Fig. 6).

## Discussion

The data presented here aimed to explore the endophytic bacterial diversity and rhizosphere soil bacterial diversity of the endangered medicinal plant *P. ludlowii*. Most previous studies on plants with Illumina amplicon sequencing have demonstrated that endophytic bacteria play an important role in plant growth (Badri et al. 2009; Müller et al. 2016; Bulgarelli et al. 2012; Weyens et al. 2009). To our knowledge, this is the first report of Illumina amplicon sequencing being used to study the endophytic bacterial community structure in the endangered medicinal plant *P. ludlowii*.

The sequence analysis revealed that endophytic bacteria were clustered into 43 phyla; Cyanobacteria was the most dominant phylum, accounting for 70.3–88.0% of all bacterial sequences in the different tissues, followed by Proteobacteria (11.9–19.6%). Cyanobacteria are the oldest photoautotrophs with nitrogen fixation capabilities and synthesize a large variety of metabolic compounds that exhibit biomaterial and biofertilizer production capabilities, including phycobiliproteins (PBPs), which may be an important target in biotechnology and biomedical research (Gonzalez et al. 2019; Rastogi and Sinha 2009; Rastogi et al. 2017). Cyanobacteria are well-known bacteria with an immense amount of pharmacologically active functions, such as antibiotic, anti-inflammatory, antioxidant and anti-Alzheimer's disease properties (Chaubey et al. 2019; Sonani et al. 2014; Singh et al. 2014). *P. ludlowii* has anti-inflammatory and antioxidative functions, while Cyanophyta is the dominant phylum in the endophytic bacterial community of *P. ludlowii*; therefore, the medicinal value of *P. ludlowii* may be associated with Cyanobacteria, and the pharmacological characteristics of Cyanobacteria may contribute to the high-quality medicinal value of *P. ludlowii*.

Proteobacteria and Acidobacteria are common microbial communities in other plants and soils (Manter et al. 2010; Bulgarelli et al. 2012; Jackson et al. 2013; Lin et al. 2019; Juan et al. 2019; Yaoben et al. 2019). Similarities were evaluated in this study. Proteobacteria and Acidobacteria were the predominant phyla in the rhizosphere soil, accounting for 21.2–31.1% and 20.9–23.6% of all bacterial sequences, respectively, while the abundance of Proteobacteria decreased with increasing soil depth. In contrast, Acidobacteria increased with increasing soil depth. It has been reported that Proteobacteria plays an important role in natural processes and has potential for application in treating wastewater, increasing tolerance to pollutants and improving the soil environment (Jeon et al. 2003; Yaoben et al. 2019; Kragelund et al. 2007). The dominant taxa in the upper soil supported the upper soil as the main site of soil organic matter degradation and soil improvement versus the other soil layers. Proteobacteria were more ubiquitous in all samples of *P. ludlowii*, and their potential function may be to regulate the immune system of *P. ludlowii* and improve the viability of *P. ludlowii* in extreme environments.

The sequences were classified into 19 different genera among all tissues and soils; however, the abundance of the bacterial communities was significantly different in all samples. Pseudomonas was the dominant genus in rhizosphere soil; however, its relative abundance decreased with soil depth, and several prior documents have shown that Pseudomonas is commonly distributed in plants and is known to have beneficial effects on plant growth and nutrient availability in addition to showing biocontrol activity against pests (Chen et al. 2014; Lee et al. 2019; Sousa et al. 2016). Nevertheless, Pseudomonas was less abundant in the roots, leaves, flowers and fruits and was even absent from the stems, which may be the reason that *P. ludlowii* is endangered. *P. ludlowii* may not provide a superior colonization environment for Pseudomonas, resulting in the lower distribution of this taxa in *P. ludlowii*, reducing the defensive abilities of the plant, affecting the absorption and utilization of nutrients, and eventually reducing the plant growth rate.

Rhizosphere microorganisms could contribute to plant health, growth and productivity (Cheng et al. 2020; Mendes et al. 2013). However, some important microbial groups, such as Pseudomonas and Bacillus, decreased with soil depth. Previous studies revealed that Bacillus potentially manipulates the host’s redox status and contributes to overcoming a critical period in development and seedling establishment due to its high catalase activities and superoxide contents (Pitzschke 2016). The dominant taxa in the upper soil supported the upper soil as a site of significant activity versus the other soil layers. The results indicated that the upper soil might play more vital roles than the middle and deep soil in improving seed germination, plant growth, and nutrient availability.

Endophytic bacteria are mainly derived from rhizosphere soil bacteria. In this study, the bacterial structure of plant tissues was significantly different, and the results were consistent with those of other studies showing that different plant tissues harbored different bacterial communities (Liu et al. 2015; Ren et al. 2019; Mûller et al. 2016). The structure of the bacterial community was similar among different deep soils; however, the bacterial structures of the roots and soil were more similar compared with those of other tissues (stem, leaf, flower and fruit), and the bacterial structure of plant tissues and soils had different levels of overlap. This indicated that endophytic bacteria might come from rhizosphere soil and be transferred from roots to other plant tissues due to the relationship between the roots and soil. Many previous studies have shown that endophytic bacteria mainly come from rhizosphere soil and are affected by soil microbes (Mûller et al. 2016; Bertollo 2001; McInory and Kloepper 1994).

The abundance of gene families in tissues and soil communities and the functional classification schemes of the KO and Clusters of Orthologs Groups (COG) databases were determined by phylogenetic investigation of communities by reconstruction of their unobserved states (PICRUSt). PICRUSt analysis showed that the gene families belonging to cellular processes, environmental information processing, genetic information processing, metabolism and organismal systems were detected among the samples. Among them, those related to metabolism were markedly most abundant. Metabolism, including energy, fatty acid, histidine, methane, nitrogen, propanoate and tryptophan metabolism, is widespread and plays extensive roles in prokaryotes. The analysis of level 3 KO groups showed that transporters were most abundant across all samples; most transporters were present in cell membranes and involved in detoxification processes, organ growth, nutrition, development and response to abiotic and biotic stresses (Do et al. 2018). The functional abundance of DNA repair and recombination proteins, ABC transporters, general function prediction, ribosomes and the two-component system was similar between the different tissues and soils, whereas the functional abundance of peptidases, photosynthesis and photosynthesis proteins was much higher in plant tissues than in soil. Overall, the functional abundance of gene families was similar, and the results indicated that there were relationships between endophytic bacteria and soil microorganisms. Thus, it is possible that *P. ludlowii* has high adaptability to local environmental conditions and has formed mutualistic associations with bacteria.
